# Supplementary material for: The Influence of Alcohol Consumption on Fighting, Shoplifting and Vandalism in Young Adults
Source: Int J Environ Res Public Health. 2021 Mar 28;18(7):3509. doi: 10.3390/ijerph18073509 (PMC8036294; doi:10.3390/ijerph18073509)
Supplement: Supplementary file 1 [file ijerph-18-03509-s001.pdf]

Supplementary Table S1. Comparison of sociodemographic and parental characteristics for the those that have consumed alcohol in the past year (sample used in analysis; $n \leq 3,408$) and those that have not consumed alcohol in the past year at age 24 years ($n \leq 166$)

	**Drinkers**	**Non-drinkers**	***p value***
**Sociodemographic characteristics**			
Age in years (mean (sd))	24.5 (0.79)	24.4 (0.78)	0.141
Male sex (% (n))	37 (1,277)	40 (66)	0.552
Non-white ethnicity (% (n))	2 (67)	5 (8)	0.010
Maternal education - high school only (% (n))	50 (1,665)	59 (93)	0.030
Housing tenure - rented (% (n))	14 (468)	26 (40)	< 0.001
Household crowding - 1+ person per room (% (n))	4 (122)	6 (10)	0.083
Maternal age at delivery in years (mean (sd))	29.4 (4.53)	29.0 (5.27)	0.216
**Parental characteristics**			
Parental crime across childhood (% (n))	13 (429)	10 (16)	0.302
Parental alcoholism across childhood (% (n))	8 (249)	4 (6)	0.082
